# Disentangling health information appraisal competence: Results from an interdisciplinary scoping review and online consultation among Swiss stakeholders

**DOI:** 10.1371/journal.pone.0235474

**Published:** 2020-07-02

**Authors:** Nicola Diviani, Jelena Obrenovic, Cassandra L. Montoya, Katarzyna Karcz

**Affiliations:** 1 University of Lucerne, Lucerne, Switzerland; 2 Swiss Paraplegic Research, Nottwil, Switzerland; University of Birmingham, UNITED KINGDOM

## Abstract

**Background:**

The ability to critically appraise health information–often referred to as critical health literacy–is recognized as a crucial component of health literacy. Yet to date, it is not clear what specific abilities are needed to adequately accomplish this task, thereby hindering both its assessment and the development and evaluation of related interventions. By systematically building on past research, this study aimed to operationally define the concept of *health information appraisal competence*.

**Methods:**

We systematically searched five scholarly databases to identify the conceptualizations and operational definitions of information appraisal in different disciplines. The resulting operationalization was subsequently validated through an online consultation exercise among 85 Swiss stakeholders.

**Results:**

Ninety-four publications were included in the review to the point of saturation. We extracted 646 skills, attitudes, and knowledge for health information appraisal. We then collated overlapping or duplicate statements, which produced a list of 43 unique statements belonging to six emergent themes or core competences: (1) basic competence, (2) predisposition, (3) identification competence, (4) critical evaluation competence, (5) selection competence, and (6) application competence. The consultation exercise enriched the operationalization of some of the core competences and confirmed the importance of all competences. Most skills, attitudes, and knowledge, however, were assigned low feasibility by the stakeholders.

**Conclusions:**

This study was the first attempt to systematically operationalize *health information appraisal competence*. From a theoretical perspective, it sheds light on an understudied, health literacy domain, thus contributing to clarity around the concept. From a practical perspective, it provides a strong theoretical basis for the development of a tool to measure *health information appraisal competence*. This could be used routinely as a screening tool, as an outcome measure for public health interventions, or to identify citizens who are most at risk. Furthermore, it will provide support for the development of future interventions to build *health information appraisal competence* in the population.

## Introduction

Due to its proven potential in mitigating several adverse health outcomes, such as lower usage of screening programs, less satisfaction with use of health services, lower adherence to medical regimens, or higher rates of hospitalization [1], health literacy (HL) is now recognized as one of the main determinants of population health. As such, enhancing HL is increasingly being included in health promotion strategies worldwide. The World Health Organization, for instance, acknowledges that HL will be crucial in determining whether the social, economic, and environmental ambitions of the 2030 Agenda for Sustainable Development will be fully realized [2].

Our understanding of the concept of HL has undergone several important changes over the past decades. When it was first introduced in the field of health education more than 40 years ago, the term merely referred to a set of technical basic skills applied to the health context [3]. Over the years, mainly ascribed to a growing interest in the topic among scholars from different disciplines, the concept has evolved to better reflect the complexity of a changing health information environment. Now, it encompasses several different competences, ranging from basic functional literacy skills, such as the ability to read and write, to more complex ones, such as the ability to navigate the healthcare system or, more recently, the ability to appraise health information.

To date, most research around HL remains almost exclusively within a functional HL domain, thus limiting our ability to conduct comprehensive assessments and to develop dedicated interventions addressing all relevant HL skills [4]. It has been argued that one of the reasons for the lack of research around other HL domains is the lack of a clear conceptualization and operational definition of these domains [5].

One clear example of this phenomenon can be illustrated by *health information appraisal competence*, which is conceptually included in the critical HL domain, originally defined by Nutbeam as the ability to critically analyze health information [6]. Although this competence is now explicitly or implicitly mentioned in most definitions of HL [7], thus far, it has been mainly studied in the context of endeavors aimed at measuring HL in its entirety as a broader construct. Recent examples are the measure developed in Japan by Ishikawa and colleagues [8], the European Health Literacy Survey [9], and the Swiss Health Literacy Survey [10]. In all of these examples, the goal of the researchers was to obtain an overall picture of people’s competences in the different HL domains and not to systematically define and/or measure each of them. Although such an approach is certainly beneficial in terms of investigating interactions among different HL domains, it also results in measures covering each domain by means of a limited number of self-report items; moreover, it does not allow a full understanding of the complexity of the individual domains [11,12]. In a few cases, critical HL has been conceptualized in a more in-depth manner in recent scholarly endeavors to better define the features that should be considered part of the concept [13,14]. While these efforts are important contributions to the conceptual clarity on what critically analyzing information means, they do not explicitly address the competences a person needs to possess in order to be health literate in this domain nor do they result in ad-hoc measures.

The recent changes in the health information context, following for instance the shift towards a more patient-centered care that requires patients to take on a more active role in decisions regarding their own health or the advent of the Internet that has provided them with an unprecedented amount of often questionable health information, have made one’s ability to appraise health information an essential competence for healthcare consumers [15]. Although the need to address critical HL, particularly, health information appraisal, has by now been recognized by both HL scholars and leading world health institutions, no systematic efforts so far have been devoted to the conceptual and operational definition of these aspects or, in other words, to understand what competences appraising health information entails [16]. This makes it almost impossible to develop a dedicated tool to assess these competences and to design dedicated theory- and evidence-based interventions to build these skills in the population.

Against this background, the overall aim of this study is to fill the conceptual gap in the HL literature highlighted in the previous paragraphs and to advance the understanding of critical HL. More specifically, it aims at identifying the range of competences (i.e., skills, knowledge, and attitudes) that have been understood in past research as components of critical appraisal of information and at completing and validating it in the context of health by involving relevant stakeholders. This will result in the first comprehensive conceptualization of *health information appraisal competence* and an inclusive list of the skills, knowledge, and attitudes that are needed to appraise health information.

## Materials and methods

### Methodological approach

To attain the aim outlined above, a scoping review of the literature was conducted. The scoping methodology was chosen because the aim of this first part of the study was not a quantitative synthesis or a quality assessment of existing evidence, but rather, the specification of a concept [17]. The scoping review process adhered to the six stages described by Arksey and O’Malley: 1) identifying the research question; 2) identifying relevant studies; 3) study selection; 4) charting the data; 5) collating, summarizing, and reporting the results; and 6) a consultation exercise [18]. The details of each stage are presented below. Every step of the process was conducted by at least two independent reviewers. Disagreements were discussed and resolved during regular meetings within the research team.

#### Stage 1. Identifying the research question

Based on the overall objective and specific aims described above, the three leading research questions of the study were:

RQ1: What are the core competences involved in information appraisal?
RQ2: What specific knowledge, skills, and attitudes do these competences entail?
RQ3: To what extent are these competences (including the specific skills, knowledge, and attitudes) relevant in the specific context of the appraisal of health information?

#### Stage 2. Identifying relevant studies

Appraisal of information has been the object of research in different disciplines, including health communication, psychology, and education. This scoping review aimed to cover the conceptualizations, operational definitions, and measures of information appraisal available in HL, information literacy, digital literacy, media literacy, critical thinking, and cognitive psychology literature. Five different scholarly databases were selected to cover the different disciplines of interest: ERIC, PsycINFO, Medline, JSTOR, and Web of Science. The databases were searched in September 2018 using a combination of search terms chosen to cover the concept of information appraisal (i.e., “information appraisal” OR “information evaluation” OR “information assessment” OR “information judgment”).

#### Stage 3. Study selection

The authors agreed that an iterative process to the exclusion, selection of studies, and data extraction was appropriate. In order to manage the large amount of documents, a hierarchy of steps for the exclusion of literature was developed. A bibliographic manager database (Zotero) supported the management of the body of literature and exclusion process. A two-phase screening process was conducted. After de-duplication, the titles and abstracts of all retrieved documents were screened to identify possible relevant literature (first phase). In this phase, documents were excluded if they: i) were not written in English, ii) did not mention information appraisal (or a variation) in the title or abstract, iii) mentioned information appraisal, but it was not performed by a human being (e.g., description of automated tools to evaluate online health information), or iv) mentioned information appraisal performed by a human being, but in the context of a specific technical information (e.g., evaluation of patient charts or appraisal of the quality of scientific articles). Selected documents were then downloaded for full-text screening (second phase). In this phase, documents were excluded if they did not mention a conceptualization, operational definition, or measure of information appraisal. No limits were set in terms of study type, publication date, and country.

#### Stage 4. Charting the data

The scoping review involved data extraction to the point of saturation when new descriptions, concepts, or components could no longer be identified. This approach, which has been adopted successfully in the past [19,20], was chosen because of a large number of documents and because our objective was neither a quantitative synthesis of evidence nor to cover all possibly relevant literature, but rather, to have a broad overview of the definitions and measures of information appraisal. The information was extracted and stored on an Excel spreadsheet for data management and to enable qualitative analysis. The final extraction table was reviewed by all of the authors. The inconsistencies noted were discussed and revisions were made. Besides the formal characteristics of the included papers—*author(s)*, *publication date*, and *discipline(s)–*all skills, knowledge, and attitudes related to information appraisal were extracted. Data synthesis was performed in two steps. In the first step, all extracted statements related to core skills, knowledge, or attitudes for information appraisal were listed and overlapping or duplicate statements were collated. This resulted in a list of unique statements. In the second step, following thematic analysis, statements were grouped into emergent themes [21]. Once it became apparent that new information could no longer be extracted, 10 more papers were reviewed and the data extracted and analyzed to ensure that the point of saturation had been reached [22].

#### Stage 5. Collating, summarizing, and reporting the results

The information from the studies was collated, summarized, and reported. The results were also used as an input for the subsequent consultation exercise, which is presented in the following paragraph.

#### Stage 6. Consultation exercise

This final step is considered optional, but it is deemed as a valuable tool to enrich the data and to increase the relevance of the results of a scoping review for policy and practice [18]. Therefore, we decided to conduct an online consultation with Swiss stakeholders aimed at enriching and validating the conceptualization resulting from the scoping review, and at confirming its validity in the specific context of health information, by including the perspective of the relevant stakeholders. We defined three groups of stakeholders that are relevant in this context: i) people working in a field related to healthcare (doctors, nurses, social workers, policy makers), ii) researchers in a health-related field (health communication, health psychology, social sciences, etc.), and iii) patients’ and consumers’ representatives. An invitation to take part in an online consultation was sent to relevant institutions (hospitals, patients’ and consumers’ associations, public health offices, universities) and selected individuals across the three language regions of Switzerland.

The consultation happened in two subsequent rounds. In the first round, after a short introduction about the aim of the study, the participants were asked in an open question format to list all of the skills, attitudes, and knowledge that in their view a citizen would need in order to be able to appraise health information. In addition, they were asked to indicate their gender, age, educational level, and profession. Finally, they were asked about their availability to be contacted for the second round of the study. All answers to the first round were collected and thematically analyzed. All new skills, attitudes, and knowledge were subsequently added to the conceptualization derived from the scoping review and sent to the stakeholders for the second round. During this round, the participants were asked to rate the importance and the feasibility of each skill, attitude, and knowledge on a five-point Likert scale (1 = Not at all important/feasible; 5 = Very important/feasible). For the feasibility rating, the participants were instructed to evaluate to which extent it is reasonable to expect a layperson to have or develop the skill, attitude, or knowledge. The average importance and feasibility scores were subsequently computed for each skill, attitude, and knowledge.

### Ethical considerations

Ethical clearance was granted by the Ethikkommision Nordwest-und Zentralschweiz (EKNZ, Project-ID: 2018–00885, cleared Oct, 24th 2018). We certify that all applicable institutional and governmental regulations concerning the ethical use of human volunteers were followed during the course of this research. No consent was obtained by the participants as all data were analyzed anonymously.

## Results

### Included studies

**Fig 1:**
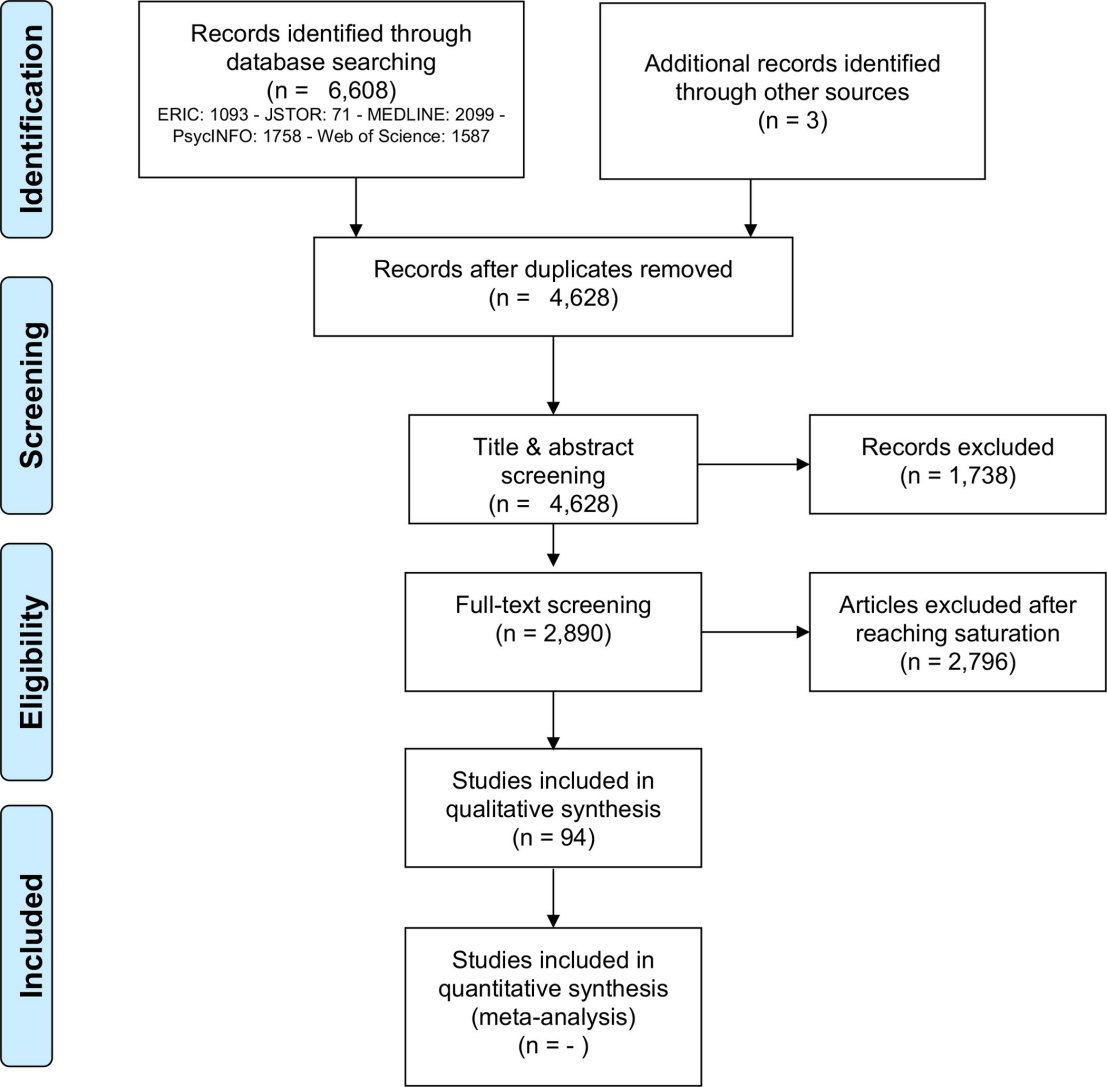
Overview of the screening process (PRISMA flowchart).

Our database search yielded a total of 6,608 documents. This was reduced to 4,628 after duplicates were removed. After reviewing the titles and abstracts from the search results, we had 2,890 potential references for data extraction. A total of 84 documents were included in the data extraction to the point of saturation, when new information was no longer obtainable. To ensure that the point of saturation had been reached, the data from 10 more documents were extracted and analyzed, for a total of 94 papers. The whole process is summarized in Fig 1.

### Characteristics of included studies

The included publications were published between 1968 and 2017. The majority were publications in the field of medicine or public health (n = 44, 46.8%), education (n = 33, 35.1%), and information and library sciences (n = 27, 28.7%). Far less represented were publications in the fields of computer science and technology (n = 15, 15.9%), psychology (n = 6, 6.4%), and media studies (n = 2, 2.1%). Several publications covered more than one discipline, e.g., those published in medical informatics journals.

### Emerging themes from the scoping review

A total of 646 statements related to information appraisal competence were extracted from the included documents. After conceptually similar statements were merged, the list was reduced into 46 unique statements. Following thematic analysis, these statements were grouped into six emergent themes, which were identified as the core competences involved in information appraisal. In the following, the six core competences will be briefly described. The complete list of skills, attitudes, and knowledge for each theme is found in Table 1.

**Table 1:** Overview of skills, attitudes, or knowledge included in the operational definition of health information appraisal competence.

	Scoping review (Study 1)	Consultation exercise (Study 2)				
		Round 1 N = 82	Round 2 N = 45			
	References	Mentioned (Yes/No)	Importance		Feasibility	
			*% (Very) important*	*% Not (at all) important*	*% (Very) feasible*	*% Not (at all) feasible*
**I. Basic competence** *A lay person should*.* *.* *.						
***a*. *Basic literacy***						
. . . be able to read and write	[23–26]	Yes	86	-	68	7
. . . be able to process and comprehend information	[23–26]	Yes	100	-	38	11
***b*. *Basic information literacy***						
. . . know how to search for and find information from different sources	[27–39]	Yes	73	4	24	22
. . . be aware that not all available information is of the same quality and applies to everyone	[34]	Yes	85	2	26	33
. . . recognize that not all information is relevant or applicable to all situations	[34]	Yes	98	2	13	33
… be familiar with the consequences of using low quality information*	-	Yes	91	-	11	43
. . . recognize that criteria exist to evaluate information and knows how to apply them**	[31,40–64]	No	78	-	13	62
***c*. *Basic health knowledge***						
… possess common knowledge of the body, health, and illnesses	[65]	Yes	79	-	37	26
… know the basics of a healthy lifestyle and relevant health behaviors	[34]	Yes	91	-	34	16
… be able to understand chances and limitations of medical and health science	[65]	Yes	58	7	11	56
**II. Predisposing factors** *A lay person should*.* *.* *.						
***a*. *Personal qualities***						
… have an open-minded attitude and be aware of own biases (e.g. preconceptions, prejudice)	[66]	Yes	66	34	7	51
… have a certain degree of trust in health care professionals and medical science*	-	Yes	75	7	29	24
… be willing to invest time in information appraisal*	-	Yes	78	2	18	44
. . . have an understanding and awareness of own body*	-	Yes	84	2	24	22
… know when to ask for professional help*	-	Yes	93	-	36	11
… be able to seek alternatives and change opinion	[67,68]	Yes	89	2	14	45
… be inquisitive and have a motivation to gain new knowledge	[66,68]	Yes	49	-	9	36
… have a sensibility for feelings, knowledge and degree of sophistication of other individuals	[68]	Yes	50	14	7	47
… take a questioning/critical attitude towards information	[23,29,35,42,69–72]	Yes	82	2	13	42
… know how to use an organized approach to problem solving**	[66,68]	No	67	9	7	53
***b*. *Cognitive skills***						
… be able to focus on the topic of interest and keep in mind the original concern**	[68]	No	60	7	11	40
… have an analytical approach towards information	[23,26,29,31,33,37,39,40,42,43,48,57,66–70,72–76,76–92]	Yes	64	13	9	64
**III. Identification competence** *A lay person should be able to…*						
***a*. *Information related***						
… isolate the individual components of information**	[23,69]	No	70	9	19	30
… identify the purpose of the information	[37,39,93]	Yes	89	-	25	14
… recognize the main message	[23,68,73,89,94]	Yes	93	4	39	13
… discern the arguments used to support the message	[23,68]	Yes	84	4	16	25
… recognize bias in the information (e.g. inferences, controversial issues, or omitted/missing information, commercial interests, assumptions, propaganda, overinterpretation)*	-	Yes	87	7	7	59
… distinguish between facts and opinions	[37,95]	Yes	82	7	11	52
***b*. *Author/source related***						
. . . identify the author/source of the information	[32,94,96]	Yes	70	5	27	39
. . . recognize the qualifications or competences of the author/source of the information	[36,47,55]	Yes	70	9	14	57
… distinguish the position, interests, and values of the author/source of the information	[30,97]	Yes	74	7	11	52
. . . identify the potential effect of the author’s position, interests, and values on the content*	-	Yes	77	5	11	57
***c*. *User-related***						
… identify the intended audience of the information**	[27,98]	No	59	11	223	32
… define the nature and extent of own information need**	[27,99]	No	73	11	11	39
**IV. Evaluation competence** *A lay person should be able to…*						
***a*. *Intrinsic quality evaluation***						
… evaluate the accuracy of the information	[100]	Yes	88	5	19	48
. . . assess the comprehensibility of the information**	[40]	No	71	9	24	22
… appraise the comprehensiveness/completeness of the information**	[41,75,93]	No	76	7	12	42
… evaluate the topicality of information**	[40,41,43,47,56,64,75,87,93,96,100]	No	81	9	33	21
… judge the overall value/quality of information	[28,34,40,41,52,70,71,81,99,101–106]	Yes	86	8	12	30
***b*. *Source-related evaluation***						
… assess the credibility (i.e., expertise, trustworthiness) of the author/source	[32,37,38,41,42,53,75–77,93,100,107–109]	Yes	88	5	19	42
… appraise the information’s risk of bias	[23,26,42,75,86,89,102]	Yes	71	10	10	61
… evaluate the authenticity of the information	[51,70,73]	Yes	72	12	12	45
***c*. *User-related evaluation***						
… estimate the relevance/usefulness of the information for own purposes	[29,36,39,65,77,78,95,100,110–112]	Yes	86	2	21	26
… estimate the applicability of the information for own purposes	[107,113]	Yes	81	5	28	26
**V. Selection competence** *A lay person should be able to…*						
***a*. *Information-related***						
. . . select the most complete information**	[75]	No	73	10	10	39
***b*. *Source-related***						
… opt for the information coming from the most credible source	[53,64,74,93]	Yes	90	-	14	43
***c*. *User-related***						
… choose the information that is most relevant**	[38,78]	No	93	-	17	38
**VI. Application competence** *A lay person should be able to…*						
… synthesize information and convey reasonable agreement or disagreement**	[40,58,69,73,77,80–82,114–116]	No	66	7	17	46
… decide whether additional information is needed	[31]	Yes	88	2	19	26
… use information to make an informed decision within a specific context or situation**	[53,66,68,69,71,79,83]	No	88	-	14	36
… determine whether the obtained information changes own practice or values**	[31]	No	71	9	12	55
… resolve contradictions when dealing with conflicting information	[25,70,78]	Yes	81	7	7	55
… compare and integrate new information with previous knowledge	[29,31,40,65,117]	Yes	78	5	21	40

* Item which was only mentioned in the consultation exercise

** Item which was only mentioned in the literature

#### Basic competence

This first health information appraisal core competence refers to all the basic skills and knowledge every person should possess prior to any information appraisal effort. This includes basic literacy skills (e.g., being able to read and write), basic information literacy skills (e.g., knowing how to search for and find information from different sources), and basic health knowledge (e.g., having a basic understanding of the body).

#### Predisposition

Predisposition includes all of the individual’s attitudes, and skills that can facilitate the process of health information appraisal, such as by making the evaluation process relevant for the individual. We distinguish between two main sub-categories: *personal qualities* and *cognitive skills*. While the first addresses personal characteristics and attitudes an individual should possess for the appraisal process (e.g., being curious or being open to new knowledge), the latter includes higher order cognitive skills, which can aid an individual in appraising information (e.g., being able to approach information in a systematic way).

#### Identification competence

This core competence refers to an individual’s ability to identify and isolate aspects of health information, which can be useful for the quality evaluation. We distinguish here between three types of aspects: those that are *information-related* (i.e., intrinsic essential aspects of information, such as its topic or level of detail), those that are *source-related* (i.e., aspects of the source of the information, such as its qualifications), and those that are *user-related* (i.e., those aspects that refer to the user of information, such as one’s own information needs).

#### Evaluation competence

This core competence refers to the ability to perform a critical evaluation of the different quality aspects of health information. Skills within this theme, together with identification and selection skills (but to a lesser extent), were the ones most frequently cited in the included studies. Similar to the previous theme, evaluation skills can be distinguished among *information-related* (e.g., evaluation of whether the information is complete), *source-related* (e.g., assessing whether the source is trustworthy), and *user-related* (e.g., appraising whether the information matches one’s own information needs).

#### Selection competence

The fifth identified core competence refers to an individual’s ability to select the most appropriate information based on the evaluation of its different information-related, source-related, and user-related aspects and to discard all information that does not fit.

#### Application competence

This last core competence refers to an individual’s ability to use the information appraised for his or her own purposes and to apply it to make informed decisions regarding his or her own health. This includes being able to decide whether the information collected is enough to make a decision or the ability to integrate newly acquired information into pre-existing knowledge.

### Consultation exercise

An invitation to take part in the online consultation exercise was sent to a total of 190 Swiss stakeholders from the three Swiss linguistic regions. Of those invited, 82 agreed to participate and filled out the first questionnaire (43% response rate). The majority of the stakeholders were female (n = 60, 74%) and ages ranged between 21 and 61 years old (mean age = 42; SD = 12). Respondents could indicate one or more professions. Most identified themselves as patients’ representatives (n = 33, 40%) or healthcare professionals (n = 33, 40%), followed by researchers (n = 20, 24%), policy makers (n = 14, 17%), students (n = 8, 10%), and educators (n = 3, 4%). Most participants to the first questionnaire agreed to be contacted again for the second round (n = 66, 81%). Forty-five of them actually took part in the second round (68% response rate).

#### First qualitative round

In the first round of the consultation exercise, the stakeholders were asked in an open question format to list all of the competences, attitudes, and knowledge that in their view a citizen would need in order to be able to appraise health information. Qualitative thematic analysis of the textual answers showed a great degree of overlap between the skills, attitudes, and knowledge mentioned by the stakeholders and those extracted from the scientific literature. More specifically, the six identified themes or core competences were shown to adequately cover all relevant aspects and to be relevant also in the specific context of health information. The analysis, however, also provided some additional information regarding the specific skills, knowledge, or attitudes that are part of these core competences. In particular, it added to the predisposing factors, highlighting the importance of having a certain degree of trust in healthcare professionals and in medical science or willingness to invest time in information appraisal. At the same time, the answers of the stakeholders seemed to assign less importance to the more “technical” aspects of appraisal, such as being able to assess the topicality of information or to identify its intended audience, compared to the literature. Skills, attitudes, and knowledge, which were only mentioned in the consultation exercise and not found in the literature, are marked with an asterisk in Table 1. Those found in the literature are marked with two asterisks.

#### Second quantitative round

In the second round of the consultation exercise, the stakeholders were presented with the full list of skills, attitudes, and knowledge extracted from the literature and derived from their answers to the open question of the first round. For each item of the list, they were asked to rate its importance for *health information appraisal competence* as well as its feasibility or, in other words, the extent to which they believe it can be reasonably expected from citizens to possess a certain skill, attitude, or knowledge.

A detailed overview of the ratings is presented in Table 1. In terms of importance, most items were rated as *important* or *very important* by a large majority of respondents, ranging from 49% for “be inquisitive and have a motivation to gain new knowledge” to 100% for “be able to comprehend and process information”. Even for the few skills, attitudes, and knowledge in the lower end of this range, the percentages of stakeholders rating them as little or not important never exceeded 14%.

A completely different picture emerged from the feasibility ratings. With a very few exceptions, for instance “being able to read and write”, which was rated as feasible or very feasible by 68.2% of the stakeholders in the sample, for most of the listed skills, competences, attitudes, and knowledge, the stakeholders seemed reluctant to express a clear judgment regarding their feasibility, as highlighted by a clear tendency to prefer the intermediate answer option “neither feasible nor not feasible”.

## Discussion

The main aim of this interdisciplinary scoping review and stakeholder consultation was to conceptualize *health information appraisal competence* and to identify and operationalize the core competences involved in the process. This was in response to a gap in the HL literature, which so far has very much focused, especially from an empirical point of view, on the functional component of the concept [118]. From theoretical and conceptual perspectives, the findings of the present study provide important new insights on the concept of HL, particularly on its critical component. On the other hand, in a more research- and practice-oriented perspective, these results provide a solid basis for the development of future research in the field. Findings from this research will in turn be greatly beneficial for both individual and public health. In the following, we will discuss our findings in terms of their significance for HL as a concept and of their implications for both research and practice.

Our findings have shown that health information appraisal is a complex concept that entails skills, attitudes, and knowledge in six interrelated but distinct areas, which we have labeled as *core competences*. First, individuals need a basic competence that are is specific to information appraisal but is necessary for the appraisal process to even begin. This include basic literacy skills (e.g., being able to read), basic level of information literacy (i.e., knowing how to navigate information), as well as basic knowledge about health and science. Second, it seems clear that people need to be predisposed for appraisal. This was shown to entail both personal qualities (e.g., being curious or having a questioning attitude) and cognitive skills (e.g., being able to focus on the different components of information or being able to adopt a structured approach to information). Third, individuals should be able to identify and isolate the aspects of the information that can be useful for the evaluation of its intrinsic and extrinsic qualities. This means, for instance, being able to identify the source of a statement or to understand what kind of information one needs. Fourth, people should be able to perform a critical evaluation of the intrinsic and extrinsic qualities of health information. This includes being able to ascertain whether the information is complete, whether its source is trustworthy, or whether it is relevant for one’s purposes. Fifth, individuals need to be able to select the information coming from the most credible source, being of the highest quality (e.g., most up-to-date and most complete) and being more relevant for one’s needs. Finally, individuals should be able to apply the selected information in order to make informed decisions. This means being able to decide whether or not more information is needed or being able to integrate the newly found information with pre-existing knowledge.

Such complexity and variety of skills, attitudes, and knowledge is not surprising. The very concept of HL, indeed, has long been recognized as a multi-faceted one [119]. This complexity, however, makes it even clearer than before that our current understanding of the critical dimension of HL, both in terms of conceptualization and of measurement, is by far not sufficient to fully capture all of the facets of the concept. At the conceptual level, except in a few cases where information appraisal has been described in more detail [13,14], information appraisal is usually only mentioned within the definitions of HL, with no identification of the specific competences it entails. The picture is not very different with regards to measurement. Traditionally used measures of HL, such as the Test of Functional Health Literacy in Adults [120], the Rapid Estimate of Adult Literacy in Medicine [121], or the Newest Vital Sign [122], do not address information appraisal at all as they only focus on the functional component of HL. However, even those measures designed to assess HL in its entirety and multi-dimensionality, such as the European Health Literacy Survey [9] or the All Aspects of Health Literacy Scale [123], do not devote ample space to the dimension of information appraisal. In most cases, only a few items are dedicated to this aspect and they mostly refer to the evaluation itself, while other competences highlighted as central in the present study are completely left out.

The mismatch between the complexity of the concept and the current conceptualizations and measurement tools has several important implications. First, it hinders a comprehensive exploration of the pathways linking HL and health outcomes. There is a whole body of research on the relationship between HL and health outcomes, which has shown that a lower level of HL is linked to several adverse health outcomes, such as lower usage of screening programs, less satisfaction with the use of health services, lower adherence to medical regimens, higher rates of hospitalization, and even earlier death [1]. Yet most of this evidence only considers functional HL, so it is not clear which health risks are associated with the deficiency in other HL domains [124]. Second, the lack of a specific measure might leave important literacy gaps unexplored and undetected. For instance, a person could have high functional HL skills, but completely lack the ability to critically appraise health information. Current instruments are not able to detect this gap in a comprehensive and actionable way [125]. Above all, without a clearer understanding and a systematic measurement of health information appraisal, it is almost impossible to design and evaluate interventions aimed at addressing people’s critical HL skills [118].

Besides shedding a new light on the existing gap in the HL literature, the findings of this study also provide us with a solid and concrete starting point to address it in future research. Our detailed operationalization of *health information appraisal competence*, which is rooted in the literature from different disciplines and that has been enriched and validated by relevant stakeholders, can be used to inform the development and validation of a measure of health information appraisal that covers all relevant aspects. Such measure would enable clinicians and public health officials to obtain a detailed picture of the *health information appraisal competence* of their patients or of the population at large and of how this impacts health outcomes. Specifically, this could mean being able to detect the specific skills, attitudes, or knowledge that are most in need of improvement and to identify the groups of the population who are most at risk in this context. In turn, this information would inform the development of dedicated interventions to build *health information appraisal competence* in the population.

We acknowledge that our study has some limitations. The first limitation is linked to the methodological approach chosen for the scoping review, namely, the choice to include articles and extract data only to the point of thematic saturation. This approach was chosen because our search terms were very broad and resulted in a high number of potentially relevant publications. Although such a methodological approach has already been used in other reviews [19,20] and is common practice in qualitative studies [22], it might limit our confidence in having a comprehensive picture of what has been said on the topic of information appraisal in the literature. Nevertheless, the substantial overlap between what was extracted from the literature and the findings from the consultation exercise makes us confident that we have covered most, if not all, relevant aspects. The second limitation is related to the consultation exercise. First, because of our recruitment strategy, our sample cannot be considered representative of the population of interest. However, the relatively large (for a qualitative study) sample and the inclusion of different professional figures (i.e., healthcare professionals, patients’ representatives, researchers, students) make us more confident in the solidity of our findings. A further limitation of the consultation exercise is its focus on Switzerland. We know that HL is highly dependent on the demands of the health system that an individual interacts with [126]. For instance, more sophisticated HL skills might be needed to navigate a healthcare system that is highly complex and has an emphasis on self-determination. Therefore, the HL skills needed in Switzerland might not be the same ones needed in other parts of the world. Given that the Swiss healthcare system is recognized for its complexity [127], we are confident that our findings can cover the variety of skills needed for health information appraisal in both complex and less complex contexts. Moreover, in this case, our confidence is further increased by the overlap between what was extracted from international literature and the findings of the consultation exercise.

## Conclusions

The ability to appraise the increasingly large amount of health information we encounter in our everyday lives is becoming more and more important and has been recognized as a priority by researchers, practitioners, and policy makers worldwide [128]. The present study responds to a clear gap in the HL literature, which so far has neglected to devote specific attention to the concept of *health information appraisal competence*, both from theoretical and empirical perspectives. By operationalizing the concept, our findings contribute to this subject’s theoretical clarity and can be used as a strong basis for the development of dedicated measurements. In turn, this could serve as a starting point for the development of theory- and evidence-based interventions to build *health information appraisal competence* in the population.

We recognize that this is an ambitious goal, as clearly indicated by the stakeholders involved in our study, who rated the likelihood that the identified skills, attitudes, or knowledge could be mastered by the public as low. However, we believe that this is very much related to the fact that such interventions, as well as evidence on their effectiveness, are scarce. Indeed, although the need to consider HL literacy as an asset and not only as a risk factor has been highlighted by prominent scholars in the field for many years, the majority of HL interventions still focus on adapting the health information environment to the limited skills of the public. Interventions aimed at building skills are rare and mostly focus on the functional component of HL.

Therefore, we are confident that a stronger theoretical understanding of the concept of information appraisal, together with a larger evidence base on its prevalence and impact, will inspire the development of new streams of research aimed at developing and evaluating the effectiveness of interventions in this context.
